# The Potential Role of PSMA-Targeted PET in Salivary Gland Malignancies: An Updated Systematic Review

**DOI:** 10.3390/diagnostics14141516

**Published:** 2024-07-14

**Authors:** Alessio Rizzo, Domenico Albano, Federica Elisei, Manuela Racca, Francesco Dondi, Salvatore Annunziata, Marco Cuzzocrea, Francesco Bertagna, Giorgio Treglia

**Affiliations:** 1 Department of Nuclear Medicine, Candiolo Cancer Institute, FPO—IRCCS, 10060 Turin, Italy; alessio.rizzo@ircc.it (A.R.); manuela.racca@ircc.it (M.R.); 2 Division of Nuclear Medicine, Università degli Studi di Brescia and ASST Spedali Civili di Brescia, 25123 Brescia, Italy; domenico.albano@unibs.it (D.A.); f.dondi@outlook.com (F.D.); francesco.bertagna@unibs.it (F.B.); 3 Division of Nuclear Medicine, Fondazione IRCCS San Gerardo dei Tintori, 20900 Monza, Italy; federica.elisei@irccs-sangerardo.it; 4 Unità di Medicina Nucleare, TracerGLab, Dipartimento di Diagnostica per Immagini, Radioterapia Oncologica ed Ematologia, Fondazione Policlinico Universitario A. Gemelli, IRCCS, 00168 Rome, Italy; salvatore.annunziata@policlinicogemelli.it; 5 Clinic of Nuclear Medicine, Imaging Institute of Southern Switzerland, Ente Ospedaliero Cantonale, 6501 Bellinzona, Switzerland; marco.cuzzocrea@eoc.ch; 6 Faculty of Biology and Medicine, University of Lausanne, 1011 Lausanne, Switzerland; 7 Faculty of Biomedical Sciences, Università della Svizzera Italiana, 6900 Lugano, Switzerland

**Keywords:** PSMA, PET, nuclear medicine, salivary gland, adenoid-cystic carcinoma, oncology, imaging

## Abstract

Background: Recent studies have suggested using positron emission tomography/computed tomography (PET/CT) with prostate-specific membrane antigen (PSMA)-targeting radiopharmaceuticals for the detection of salivary gland malignancies (SGM), particularly adenoid-cystic carcinoma (ACC). Methods: The authors conducted an extensive review of the scientific literature to examine the potential diagnostic role of PET/CT using PSMA-targeting radiopharmaceuticals in salivary gland malignancies (SGMs) and adenoid cystic carcinoma (ACC). This study included newly diagnosed SGM patients and those with disease recurrence in their imaging evaluation. Results: This updated systematic review included a total of six studies that examined the diagnostic performance of PSMA-targeted PET/CT in ACC. The articles provided evidence of a high detection rate of PSMA-targeting PET/CT in ACC across all clinical contexts examined. SGMs other than ACC exhibited poorer diagnostic performance. Conclusions: PSMA-targeted PET/CT seems promising in detecting ACC lesions; moreover, PSMA appears to be a suitable potential target for radioligand therapy. Prospective multicentric studies are warranted to strengthen the role of PSMA-targeting radiopharmaceuticals in ACC, as both diagnostic and theragnostic agents.

## 1. Introduction

Salivary gland malignancies (SGMs) are uncommon tumors in the head and neck region. To date, twenty-four different forms of SGMs have been recognized, with mucoepidermoid and adenoid cystic carcinoma (ACC) being the most common subtypes [1]. SGMs typically arise from the parotid, submandibular, and sublingual glands [2]. A slow growth rate and a non-aggressive nature usually distinguish them. However, they show a propensity to relapse repeatedly and develop distant metastases in various anatomic sites, including the lymph nodes, lungs, and liver [3].

From a histological perspective, ACC is characterized by the presence of tubular, cribriform, and solid patterns. It is well-acknowledged that a solid growth pattern is indicative of a higher tumor grade and a more unfavorable prognosis [4]. The characteristics of this condition include a steady increase in size, extensive infiltration, frequent reappearance in the same area, and a relatively high probability of spreading to other organs [5]. While surgery and subsequent radiation therapy have enhanced survival rates in both the early and late stages of cancer progression, there is still no agreement on the most suitable systemic treatment for recurrent or metastatic illness [6]. Hence, it is imperative to promptly and quickly diagnose patients with SGMs, accurately determine the extent of the disease, and administer adequate therapy to control the disease and successfully improve the prognosis.

The standard diagnostic evaluation of SGMs involves the use of magnetic resonance imaging (MRI) and computed tomography (CT) [7]. In addition, the use of 2-[^18^F]-fluorodeoxyglucose ([^18^F]FDG) positron emission tomography (PET) can significantly improve the evaluation of metastatic lesions, hence impacting treatment decisions in around 12.5% of patients [8].

Recent immunohistochemical (IHC) and PET imaging studies have demonstrated increased prostate-specific membrane antigen (PSMA) expression in ACC [9]. This discovery indicates that the use of PSMA-targeted imaging might improve the precision of the staging process. Additionally, binding beta or alpha emitters to PSMA-targeting molecules allows radioligand therapy (RLT) in a theragnostic configuration [10].

This updated systematic review intends to collect evidence on the diagnostic utility of PSMA-targeted PET in patients with SGMs.

## 2. Materials and Methods

### 2.1. Protocol

The present systematic review was conducted following a predefined protocol [11], and the “Preferred Reporting Items for a Systematic Review and Meta-Analysis” (PRISMA 2020 statement) was used as a benchmark in its production [12]. The complete PRISMA checklist is accessible in the Supplementary Materials (Table S1). The authors declare that pre-registration of the protocol was not performed (this is feasible according to point 24 of the PRISMA checklist).

As the first step, a straightforward review question was created: “What is the diagnostic role of PSMA-targeted PET imaging in patients with salivary gland malignancies?”

A literature search according to the Population, Intervention, Comparator, Outcomes (PICO) framework was performed, establishing criteria for study eligibility as follows: patients diagnosed with SGM (Population) and undergoing PSMA-targeted PET (Intervention) as opposed to conventional imaging (Comparator); the investigated main outcome was the diagnostic performance of PSMA-guided PET imaging in SGM. Secondary outcomes were the uptake of PSMA-targeted radioligands in the SGM primary tumor and metastatic lesions and the comparison between PSMA-targeted PET and other imaging methods.

To avoid possible biases, two reviewers (A.R. and G.T.) independently performed the literature search, the selection of the studies, the data extraction, and the quality assessment. An online consensus meeting (date: 6 June 2024) solved any discrepancies among the reviewers.

### 2.2. Literature Search Strategy and Information Sources

After defining the review question, a comprehensive literature search was performed using three electronic bibliographic databases (PubMed/MEDLINE, Embase, and Cochrane Library) to search for studies evaluating the diagnostic performance of PSMA-targeting PET in SGM. Furthermore, the authors consulted the clinicaltrials.gov database to search for ongoing trials in the field of interest.

Taking into account the review question, a search algorithm based on a combination of these terms was utilized: (A) “positron” OR “PET” AND (B) “PSMA” AND (C) “salivary” OR “adenoid”. To increase the sensitivity of the literature search, no restrictions were applied regarding the year of publication or article language. Additionally, the references of the retrieved studies were also screened to find additional eligible articles in order to refine the research. The literature search was last updated on 4 June 2024.

### 2.3. Eligibility Criteria

The eligibility criteria were chosen taking into account the review question. Clinical studies reporting diagnostic information concerning the employment of PSMA-targeted PET in the staging and restaging of SGM were deemed eligible for inclusion in this systematic review. Exclusion criteria for the systematic review (qualitative analysis) were reviews, letters, comments, editorials on the topic of interest, case reports, or small case series (fewer than five enrolled patients) on the analyzed topic (as these articles are characterized by poor-quality evidence and are typically affected by publication bias), as well as original articles dealing with different fields of interest (including pre-clinical studies).

### 2.4. Selection Process

Based on the predefined inclusion/exclusion criteria and the literature search strategy, the titles and abstracts of the gathered papers were reviewed. Moreover, the reviewers decided on whether to include or exclude the screened studies in this review, specifying the reason.

### 2.5. Data Collection Process and Data Extraction

Data from eligible studies were extracted and collected, taking advantage of the information in the full text, tables, figures, and Supplementary Materials. For each study included in this systematic review, the following data were extracted and collected using predefined data collection forms: general study information (authors, publication year, country, study design, and funding sources); patient characteristics (sample size, age, sex ratio, clinical setting, and other diagnostic imaging); index text characteristics (type of PSMA-radioligand employed, type of hybrid imaging protocol, patient preparation, radiopharmaceutical administered activity, uptake time between PSMA-radioligand administration and image acquisition, and the protocol for image analysis), data on the diagnostic performance of PSMA-targeted PET in SGM on a per-patient-based analysis, and the diagnostic benchmark used.

### 2.6. Quality Assessment (Risk of Bias Assessment)

The selected method used for assessing the risk of bias in individual studies and the applicability to the review question was QUADAS-2, a tool for evaluating quality in diagnostic test accuracy studies [13]. Four domains (patient selection, index test, reference standard, and flow and timing) were assessed regarding the risk of bias, and three fields were evaluated regarding applicability (patient selection, index test, and reference standard).

## 3. Results

### 3.1. Literature Search and Study Selection

**Figure 1:**
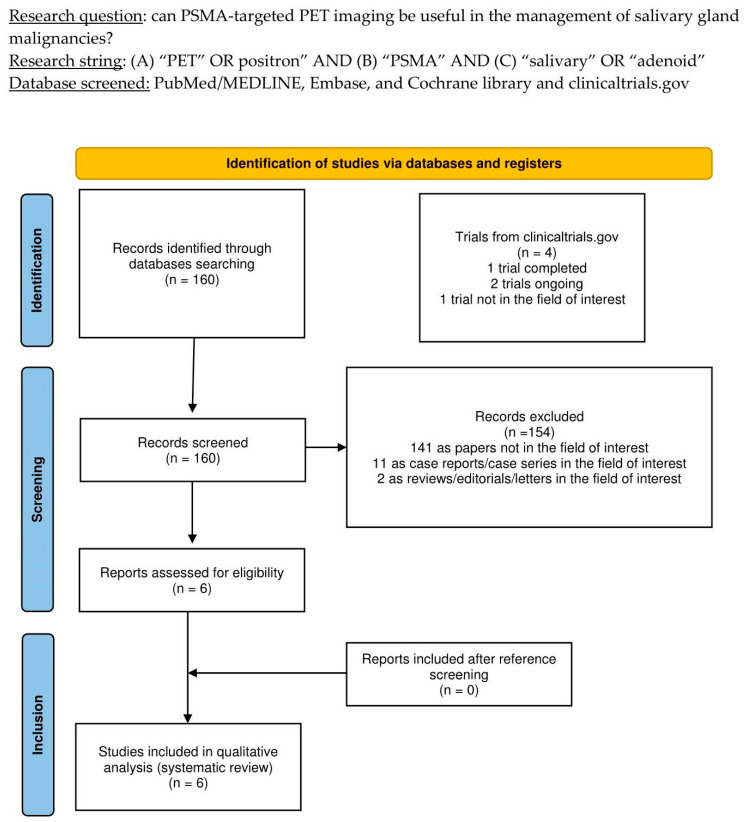
PRISMA flow-chart summarizing the study selection process. PRISMA: Preferred Reporting Items for Systematic Reviews and Meta-Analysis.

The thorough literature search yielded 160 records. According to the information in Section 2, these 160 publications were scrutinized for eligibility based on preconceived criteria for inclusion and exclusion, and 154 documents were excluded (due to being unrelated to the topic of interest, case reports, or reviews). The six remaining studies were assessed as suitable for inclusion in this systematic review (qualitative synthesis). After screening the included articles’ references, no additional studies were deemed eligible for inclusion in this systematic review [14,15,16,17,18,19]. Figure 1 summarizes the study selection process.

### 3.2. Study Characteristics

The six studies that met the criteria for inclusion in this systematic review (qualitative analysis), which included a total of 115 SGM patients, are thoroughly analyzed in Table 1, Table 2 and Table 3. The selected studies were published from 2017 to 2024 in the Netherlands (3/6), China (1/6), Germany (1/6), and India (1/6). Half of the included papers used prospective designs [15,17,19], while the other half retrospectively analyzed their casuistries [14,16,18]. All of the included trials were single-center studies [14,15,16,17,18,19]; moreover, three included papers disclosed financing in their text [15,17,18].

According to Table 2, the number of enrolled SGM patients in each study ranged from six to 30; their average age ranged from 39 to 70 years, and the percentage of men varied from 39% to 84%. In all the articles included, the index test was mainly employed for restaging SGM patients; however, in three studies, a minor subset of patients undergoing disease staging was included [17,18,19]. All publications enlisted ACC in their casuistries concerning histologic subtypes, enrolling 99 patients. Among them, three studies accounted for the enrollment of other subtypes of SGM, namely salivary ductal carcinoma, acinic cell carcinoma, and adenocarcinoma [15,16,18]. Concerning the location of primary tumors, the reported anatomic sites were major salivary glands, such as parotid and submandibular glands, sublingual glands, the palate, the nasal cavity, and cheek mucosa [14,15,16,17,18,19]. Additional sites of primary ACC, namely the trachea, bronchus, and Bartholin gland, were described in one study [15]. Finally, three included papers compared the index test outcomes with [^18^F]FDG PET [14,17,19]; one used solely CT [18] and one added MRI [15] as a comparative imaging technique. The remaining study did not compare PSMA-targeted imaging with any morphological or functional imaging technique. Table 2 presents all tumor locations, pathology, and comparative imaging data.

Based on the constrained number of included papers, the index test characteristics did not vary significantly between the included studies, as shown in Table 3. Four included studies administered [^68^Ga]Ga-PSMA-11 as a PSMA-targeting compound for PET imaging [14,15,16,19]; however, one of these studies administered [^18^F]PSMA-1007 in a subset of patients [18]. Finally, in one study, the radiopharmaceutical involved in the study design was [^68^Ga]Ga-PSMA-617 [17]. All the studies, except for one, reported the administered radiopharmaceutical activity using relative values ranging from 1.8 to 3.0 MBq/Kg [14,15,16,17,19]. The only study reporting the administered activities in absolute values declared an average of 116 MBq for [^68^Ga]Ga-PSMA-11 and 281 MBq for [^18^F]PSMA-1007 [18]. The time between radiopharmaceutical administration varied from 50 to 63 min for renally excreted PSMA-targeting radiopharmaceutical forms and was 98 min for the subgroup of patients undergoing PET imaging with [^18^F]PSMA-1007. All the included studies accounted for a qualitative and semiquantitative evaluation of PET imaging involving the calculation of the lesions’ SUV_max_ in their study design [14,15,16,17,18,19]. Moreover, two studies reported the values of TBR, using healthy parotid, liver, muscle, kidney, and blood pools as background regions [15,19].

### 3.3. Risk of Bias and Applicability

**Figure 2:**
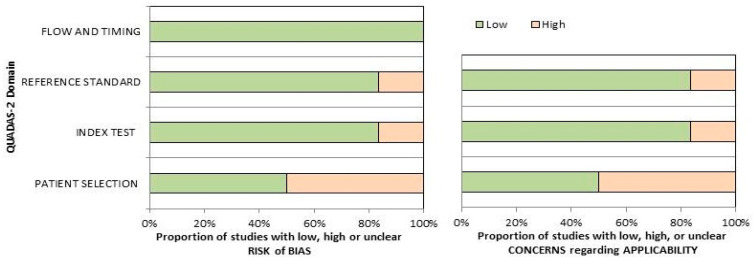
Summary of the quality assessment carried out using the QUADAS-2 tool. The authors classified the papers included in the systematic review according to their degree of bias or applicability issues for specific subjects indicated on the ordinate axis. On the other hand, the abscissa axis displays the ratio of studies.

The overall assessment of the risk of bias and concerns about the applicability of the included papers according to QUADAS-2 is provided in Figure 2. The quality assessment of the included studies revealed a significant risk of bias in the “patient selection” domain, related to the low number of the reported sample size and the inclusion of patients in different clinical settings in single trials.

### 3.4. Results of Individual Studies

The overall evaluation of PSMA-targeted PET/CT in detecting ACC lesions assessed an optimal diagnostic performance in all the included papers, especially on a per-patient-based analysis in all the investigated clinical settings, without significant differences between PET/CT scans performed in a staging or restaging setting [14,15,16,17,18,19]. This statement is corroborated by the high detection rates reported based on the per-patient-based analyses developed in the included studies, ranging from 89 to 100%, revealing metastatic sites of disease in the lymph nodes, lungs, bone, liver, peritoneum, meninges, brain, and subcutaneous adipose tissue. Conversely, the only study to perform a separate sub-analysis for SGM tumors other than ACC reported a lower detection rate of 40% [15].

None of the analyzed papers reported adverse effects from administering [^18^F]F- or [^68^Ga]Ga-labelled PSMA-targeting radiopharmaceuticals before PET imaging [14,15,16,17,18,19].

When employed in ACC diagnostics, PSMA-targeting radiopharmaceuticals showed a remarkably variable uptake (in terms of SUV_max_) in the primary tumor, local recurrence, and distant metastases; however, it was higher than the background in most of the included investigations [14,15,16,17,18,19]. In particular, the reported SUV_max_ values ranged from 2.04 to 30.1 in primary tumors, local recurrences, and distant metastases; however, significantly lower uptake values were reported in SGMs other than ACC [14,15,16,17,18,19].

When reported, PSMA-targeted PET led to an upstaging of the disease in all the evaluated clinical settings, ranging from 10% to 44% of the enrolled patients in four included studies [14,15,18,19]. Conversely, none of the patients diagnosed with SGMs other than ACC underwent upstaging after PSMA-targeted imaging [15].

Three of the included studies performed an immunohistochemistry analysis on biopsied primary or metastatic ACC, revealing remarkable inhomogeneity in PSMA expression in lesion specimens, with PSMA staining ranging from 0% to 90% [14,15,16]. Interestingly, PSMA was always expressed on the ACC cell membrane and not the tumor-associated neovasculature. When reported, PSMA staining in immunohistochemistry analysis did not correlate with PSMA radioligand uptake on PET images [14,15,16,17,18,19]. Concerning SGMs other than ACC, PSMA expression was reported in the tumor-associated neovasculature of eight out of nine samples and was not observed on the tumor’s cell membrane [15].

Finally, half of the included trials administered [^177^Lu]Lu-labelled PSMA-targeting radiopharmaceuticals in a subset or all of the enrolled patients [16,17,18]. Concerning the safety of this radiopharmaceutical, the main adverse effects encountered were grade 2 anemia, grade 3 thrombocytopenia, and mild transitory hepatic insufficiency. Moreover, a subset of patients had a subjective response of evident relief of tumor symptoms after the first cycle; the most common improvement was a reduction in pain, followed by a decrease in fatigue, less dyspnea, and improvements in facial expression due to a diminution of facial nerve palsy. Regarding the efficacy of [^177^Lu]Lu-labelled PSMA-targeting radiopharmaceuticals in ACC, only several (2/9) patients experienced a long-term response. At the same time, three had to discontinue their treatment because of disease progression, and the remaining patients had disease relapse a few months after the treatment protocol ended.

Since the included studies reporting PSMA-targeted PET/CT detection rate in ACC used different modalities for their assessment and other benchmarks to calculate it, a meta-analysis could not be accomplished [14,15,16,17,18,19]. The main results of the included papers, including semiquantitative metrics (SUV_max_), detection rate, and data concerning the number of upstaged patients, are synthesized in Table 4.

## 4. Discussion

Based upon PSMA overexpression on prostate cancer cells, radiopharmaceuticals targeting this transmembrane protein became a consolidated compound to improve the performances of molecular imaging and to develop new therapeutic instruments through radioligand therapy (RLT) in metastatic castration-resistant prostate cancer [20,21]. Furthermore, recent shreds of evidence highlighted PSMA overexpression in the neovasculature of various types of cancer other than prostate cancer, including clear cell renal carcinoma, thyroid cancer, and hepatocellular carcinoma [22,23,24]. Further immunohistochemistry studies observed how PSMA expression could regulate tumor cell invasion and neoangiogenesis by transducing integrin signals in the endothelium [25]. Building upon this rationale, scholars tried to assess the diagnostic performance of PSMA-targeted PET imaging in different types of neoplasms, including SGMs and especially ACC, in various clinical settings, such as staging in newly diagnosed patients and restaging in patients undergoing relapse of their disease [14,15,16,17,18,19]. This updated systematic review attempted to gather all the evidence concerning this emerging topic, avoiding potential sources of bias.

Although the available evidence is still weak, and the studies screened employed different methods for estimating the detection rate, all the scholars assessing the accuracy of PET/CT with PSMA-targeting radiopharmaceuticals in ACC patients found excellent results in newly diagnosed patients, as well as those who had been previously treated and had undergone disease restaging [14,15,16,17,18,19].

When compared to [^18^F]FDG PET/CT, PSMA-targeted emissive imaging showed slight superiority in the detection of oligometastatic, multifocal, or disseminated disease, being able to also detect intracranial lesions in the brain and meninges (sites of disease that are hard to evaluate with [^18^F]FDG PET/CT due to physiological high glucose metabolism in the brain), leading to upstaging for a significant number of patients. Furthermore, PSMA-targeted PET/CT revealed higher tumor uptake and a more considerable tumor boundary than [^18^F]FDG PET/CT, which may be a potential further advantage over metabolic imaging [14,17,19]. Although PSMA-guided PET showed superior diagnostic performance compared to other diagnostic techniques, one study observed a relatively poor accuracy in assessing lung metastases [17]. This observation might be explained by an insufficient accumulation of PSMA radioligands in sub-centimetric lung lesions; in addition, it can be hypothesized that ACC lung lesions harbor abundant mucinous secretions inside their lumens, which could lead to a relatively low cell density and reduced PSMA expression [26]. Furthermore, chronic lung inflammation processes might contribute significantly to this phenomenon. These observations imply that PSMA-targeted PET/CT might be a valuable instrument in disease extent assessment and the treatment planning of metastases-directed therapy in oligo-metastatic patients. However, since the gathered evidence is limited to proof-of-concept trials, more prospective multicentric studies are needed to confirm these postulations and develop shared operative guidelines.

Recently, there has been a steady rise in clinical research focusing on the use of fibroblast activation protein (FAP)-targeted PET imaging in various cancer types [27]. This developing research offers vital insights into the possible applications of this innovative imaging technology. Furthermore, recent studies have shown that FAP-targeted PET has yielded exceptional outcomes in detecting many types of cancers, including tumors usually linked to insignificant amounts of [^18^F]FDG uptake [28,29,30]. To date, a single investigation has tried to assess the possible application of PET imaging using FAP-targeting radiopharmaceuticals in ACC [31]. The study found that PET imaging outperformed contrast-enhanced CT in detecting early lesions and local and distant metastases [31]. Given the lack of any existing literature comparing these two molecular imaging procedures and the recent development of bispecific tracers that target both FAP and PSMA, it is necessary to conduct prospective studies involving both diagnostic methods to determine which instrumental examination is more reliable for this type of malignancy [32].

In the context of ACC diagnosis, it is essential to recognize the presence of one more tracer, [^11^C]methionine. This specific radiopharmaceutical allows the investigation of amino acid metabolism in different types of benign and malignant diseases, particularly ACC, demonstrating greater efficacy in well-differentiated histotypes [33,34]. However, due to the physical features of [^11^C], its utilization is constrained by the unfeasibility of large-scale manufacturing. To the best of our knowledge, no reports have compared PSMA-targeted PET and amino acid-based imaging in SGM cohorts.

Among the papers in the present systematic review, three included the administration of [^177^Lu]Lu-PSMA-targeting radiopharmaceuticals in their design [16,17,18]. Due to the constrained number of patients submitted to RLT and the absence of a comparator to assess changes in progression-free and overall survivals, drawing conclusions concerning its efficacy is not feasible. However, it is noteworthy that most patients undergoing RLT showed a clinical response and symptom relief without significant adverse effects.

One previously published review explored the potential role of PSMA-targeted PET/CT in ACC patients [35]; however, it included case reports that are characterized by low-quality evidence and affected by publication bias. The primary objective of our systematic review was to conduct an updated literature search following the PRISMA guidelines and adhering to a rigorous methodology, applying suitable standards. Additionally, case reports were excluded from our analysis due to their potential for introducing bias. Consequently, additional information was presented and discussed in our literature analysis.

Concerning the limitations and biases of this systematic review, only half of the included studies had a prospective design; moreover, all the trials enrolled a restricted number of patients. Furthermore, we observed a significant heterogeneity among the included studies concerning their methodology, patient, and index test characteristics, as well as the reference standard used to demonstrate the clinical effectiveness of the proposed diagnostic technique.

## 5. Conclusions

The qualitative data provided by this systematic review enhance the promising role of PSMA-targeted PET/CT in patients diagnosed with ACC and its potential as a theragnostic agent. Nevertheless, the authors warrant more studies to encourage a better understanding of using PSMA-targeting radiopharmaceuticals in ACC as diagnostic and theragnostic agents and its integration with conventional imaging in different clinical settings.

## Figures and Tables

**Table 1:** General study information.

First Authors [Ref.]	Year	Country	Study Design/Number of Involved Centers	Funding Sources
Kein Nulent et al. [14]	2017	The Netherlands	Single center/Retrospective	None declared
Van Boxtel et al. [15]	2020	The Netherlands	Single center/Prospective	Dutch Salivary Gland Cancer Platform and the ACC Research Foundation
Klein Nulent et al. [16]	2021	The Netherlands	Single center/Retrospective	None declared
Wang et al. [17]	2022	China	Single center/Prospective	Several funding sources disclosed
Civan et al. [18]	2023	Germany	Single center/Retrospective	Several funding sources disclosed
Shamim et al. [19]	2024	India	Single center/Prospective	None declared

**Table 2:** Patient key characteristics and clinical settings.

First Authors [Ref.]	Sample Size (No. of Patients)	Mean/Median Age (Years)	Gender (Male %)	Clinical Setting (No. Patients)	Location of Primary Tumor (No. of Patients)	SGM Subtype (No. of Patients)	Comparative Imaging
Kein Nulent et al. [14]	9	Median: 54	44%	9 Restaging	4 Parotid 3 Palate 2 Nasal cavity	9 ACC	[^18^F]FDG PET/CT; CT
Van Boxtel et al. [15]	25	Median ACC: 58 Median SDC: 70	84%	25 Restaging	22 Salivary gland 1 Trachea 1 Bronchus 1 Bartholin gland	15 ACC 10 SDC	CT; MRI
Klein Nulent et al. [16]	6	Median: 39	50%	6 Restaging	3 Parotid 1 Submandibular 1 Hard palate 1 Cheek mucosa	4 ACC 1 Adenocarcinoma 1 Acinic cell carcinoma	Not available
Wang et al. [17]	30	Mean: 43	50%	1 Staging 29 Restasging	n.a.	30 ACC	[^18^F]FDG PET/CT
Civan et al. [18]	28	Median: 59	39%	2 Staging 26 Restaging	8 Parotid 7 Submandibular 3 Sublingual 10 Minor SG	24 ACC 2 Adenocarcinoma 1 Acinic cell carcinoma 1 Sebaceous carcinoma	CT
Shamim et al. [19]	17	Mean: 44	41%	4 Staging 13 Restaging	5 Paranasal sinuses 5 Hard palate 3 Parotid 2 Submandibular 1 Nasopharynx 1 Oropharynx	17 ACC	[^18^F]FDG PET/CT

Legend: ACC: adenoid-cystic carcinoma, CT: computed tomography, FDG: fluorodeoxyglucose, MRI: magnetic resonance imaging, PET: positron emission tomography.

**Table 3:** Index test key characteristics.

First Authors [Ref.]	Tracer	Hybrid Imaging	Administered Activity	Uptake Time (Minutes)	Image Analysis
Kein Nulent et al. [14]	[^68^Ga]Ga-PSMA-11	PET/CT	2 MBq/kg	60	Qualitative; Semiquantitative (SUV_max_)
Van Boxtel et al. [15]	[^68^Ga]Ga-PSMA-11	PET/CT	3 MBq/kg	60	Qualitative; Semiquantitative (SUV_max_; TBR)
Klein Nulent et al. [16] *	[^68^Ga]Ga-PSMA-11	PET/CT	2 MBq/kg	60	Qualitative; Semiquantitative (SUV_max_)
Wang et al. [17] **	[^68^Ga]Ga-PSMA-617	PET/CT	1.8–2.2 MBq/kg	50-60	Qualitative; Semiquantitative (SUV_max_)
Civan et al. [18] **	[^68^Ga]Ga-PSMA-11 [^18^F]PSMA-1007	PET/CT	[^68^Ga]Ga-PSMA-11: 116 MBq [^18^F]PSMA-1007: 281 MBq	[^68^Ga]Ga-PSMA-11: 63 [^18^F]PSMA-1007: 98	Qualitative; Semiquantitative (SUV_max_)
Shamim et al. [19]	[^68^Ga]Ga-PSMA-11	PET/CT	111–185 MBq	45	Qualitative; Semiquantitative (SUV_max_; TBR)

* The study design involved the administration of [177Lu]Lu-labelled PSMA-targeting radiopharmaceuticals in a subset of the enrolled patients. ** The study design involved the administration of [177Lu]Lu-labelled PSMA-targeting radiopharmaceuticals in all the enrolled patients. Legend: CT: computed tomography, PET: positron emission tomography, PSMA: prostate-specific membrane antigen, SUV: standard uptake value, TBR: tumor-to-background region.

**Table 4:** Outcomes of the included studies.

First Authors [Ref.]	Disease Sites Other than SG.	Lesions SUV_max_	Immunohistochemistry for PSMA	Detection Rate	Stage Migration (No. Patient)	Outcome
Klein Nulent et al. [14]	Lymph node Lung Bone Liver Peritoneum Meninges	Different sites ranging from 2.04 to 12.97	Staining on tumor cells ranging from 5 to 90%	Per patient: 89%	Upstaging 4/9	PSMA-PET could detect local recurrence and distant metastases in ACC patients.
Van Boxtel et al. [15]	Lymph node Lung Pleura Pericardium Bone Liver Brain Subcutaneous	Different sites ranging from 0.3 to 30.2	ACC: tumor cells ranging from 0 to 90%; tumor-associated neovasculature: 0/11 samples. SDC: tumor cells 0%; tumor-associated neovasculature: 8/9 samples.	Per patient ACC: 93% Per patient SDC: 40%	Upstaging: 2/15 ACC; 0/15 SDC	PSMA-PET added diagnostic value in some of the ACC patients compared to CT.
Klein Nulent et al. [16]	Lymph node Lung Bone Liver	Different sites ranging from 3.5 to 12.5	Staining for PSMA ranging from 5% to 95%	Per patient: 100%	Not available	When PSMA expression is sufficient, PSMA-targeted RLT may improve symptoms and induce a PR or SD in one-third of patients.
Wang et al. [17]	Lymph node Lung BoneLiver	Primary tumor: 9.8 Local recurrence: 10.4 Distant metastases: 4.1	Not available	Per patient: 90%	Not available	PSMA-PET is a valuable tool for diagnosing and staging ACC. Combined with FDG PET, it can improve diagnostic performance.
Civan et al. [18]	Lymph node Lung Bone	not available	Not available	Per patient: 96%	Upstaging: 3/28	PSMA PET/CT upstaged about one-third of SGM patients compared to CT.
Shamim et al. [19]	Lymph nodes Lung Bone Liver Brain Bone	Primary tumor: 4.8 Different sites ranging from 1.8 to 8.1	Not available	Pet patient: 100%	Upstaging 2/17	PSMA PET/CT may have a theragnostic role in ACC patients and can detect additional metastatic sites compared to FDG PET.

Legend: ACC: adenoid cystic carcinoma; CT: computed tomography; FDG: fluorodeoxyglucose; PET: positron emission tomography; PSMA: prostate-specific membrane antigen; PR: partial response; RLT: radioligand therapy; SD: stable disease; SGM: salivary gland malignancy.

## Data Availability

The data presented in this study are available on request from the corresponding author.

## Supplementary Materials

The following supporting information can be downloaded at https://www.mdpi.com/article/10.3390/diagnostics14141516/s1, Table S1: PRISMA checklist.
